# The interaction between uric acid and high-density lipoprotein cholesterol on the prognosis of patients with acute myocardial infarction

**DOI:** 10.3389/fcvm.2023.1226108

**Published:** 2023-07-10

**Authors:** Yu Yang, Jian Zhang, Lin Jia, Jiannan Su, Mengqing Ma, Xianhe Lin

**Affiliations:** Department of Cardiology, The First Affiliated Hospital of Anhui Medical University, Hefei, Anhui, China

**Keywords:** high-density lipoprotein cholesterol, uric acid, uric acid to HDL cholesterol ratio, acute myocardial infarction, prognosis

## Abstract

**Background:**

The significance of uric acid (UA) and high-density lipoprotein cholesterol (HDL-C) in the prognosis of acute myocardial infarction (AMI) remains controversial. This study investigated the effect of the interaction between UA and HDL-C on the prognosis of patients with AMI.

**Methods:**

In total, 480 patients with AMI were included in this study. Baseline and follow-up data were collected, and the primary endpoint was major adverse cardiovascular events (MACE). The secondary endpoint was all-cause death. Both additive and multiplicative interactions were calculated to evaluate their interaction with prognosis. Then, the impact of UA and HDL-C ratio (UHR) on prognosis was assessed.

**Results:**

Over a median follow-up period of 41 (30,46) months, 136 (28.3%) MACEs, and 44 (9.2%) deaths were recorded. There was a positive additive interaction between UA and HDL-C for MACEs. The attributable proportion (AP) showed that 46% of the estimated effect (MACE in patients) was attributable to this interaction. The synergy index (SI) was 2.04 (1.07,3.88) for MACE, indicating that the risk for patients presenting with both risk factors was greater than the sum of the risk factors alone. Multivariate Cox regression analysis revealed that UHR independently predicted MACEs and mortality. Kaplan–Meier survival curves according to tertiles of UHR showed statistically significant differences in MACE (log-rank test, *P* < 0.001). Receiver operating characteristic (ROC) analysis showed that the area under the curve (AUC) of UHR for predicting MACE was 0.716.

**Conclusion:**

The coexistence of high UA and low HDL-C has a synergistic effect and provides further information for risk stratification of patients with AMI. UHR is a simple and easily available prognostic indicator independent of traditional risk factors.

## 1. Introduction

Acute myocardial infarction (AMI) is the leading cause of cardiovascular morbidity and mortality globally (1). Moreover, patients with AMI are prone to recurrent cardiovascular events (CVEs) despite following the treatments recommended by the current guidelines (2). Therefore, early detection and intervention for residual risk in patients with AMI are important to improve prognosis.

Uric acid (UA) is a biomarker easily obtained in clinical practice. UA is a potent oxygen scavenger, and its antioxidant effects may prevent metabolic disorders (3). However, excessive UA can damage multiple organs by inducing excessive inflammatory responses (4). It can also promote oxidative modification of low-density lipoprotein cholesterol (LDL-C) to exacerbate atherosclerosis, which is closely related to the development and progression of coronary artery disease (CAD) (5). It has been found that serum UA levels are associated with the prognosis of AMI, but the relationship remains controversial in patients with different characteristics, especially those with metabolic abnormalities (6, 7).

High-density lipoprotein cholesterol (HDL-C), an important component of lipid metabolism, has previously been considered to protect against coronary atherosclerosis (8). Emerging studies have reported that HDL-C is not always protective against CAD, and very high levels may be detrimental (9, 10). Controversy remains regarding HDL-C and cardiovascular disease (CVD) risk and prognosis, which requires further mechanistic and clinical studies.

Mechanistic studies have suggested that low serum HDL-C levels and hyperuricemia may have synergistic adverse effects on the cardiovascular system through insulin resistance and oxidative damage to endothelial cells (11–13). Recently, Hu et al. reported that elevated serum UA levels influence the effect of HDL-C on carotid atherosclerosis (14). Although an association between hyperuricemia and low HDL-C levels has been observed in cardiometabolic diseases, the effect of the interaction between UA and HDL-C on the prognosis of patients with AMI has not been adequately researched. In resource-limited settings, interaction analyses can identify patients who would benefit the most from a given intervention.

UA to HDL-C ratio (UHR), a newly proposed index of metabolism and inflammation, is associated with glucolipid metabolism-related diseases and prognosis (15–17). However, limited studies have examined the prognostic value of UHR in patients with AMI. In this study, we aimed to investigate the effect of the interaction between UA and HDL-C on the prognosis of AMI. Based on the previous analysis, we investigated whether UHR is a reliable prognostic predictor of clinical outcomes of AMI.

## 2. Methods

### 2.1. Study subjects

This study is a retrospective observational cohort study. From August 2018 to December 2019, a total of 564 patients hospitalized at the First Affiliated Hospital of Anhui Medical University for AMI were enrolled. This included patients with ST-segment elevation myocardial infarction (STEMI) or non-ST-segment elevation myocardial infarction (NSTEMI). The exclusion criteria were: malignant tumors (*n* = 19); acute or chronic infectious diseases (*n* = 33); severe cerebrovascular accidents (*n* = 9); other cardiac diseases (heart valve diseases, myocardial diseases) (*n* = 14); and patients with incomplete data (*n* = 9). AMI was defined using the Third Universal Definition (18). Ultimately, 480 patients were included, including 351 men aged 27–93 years. The study was approved by the ethics committee (approval no. PJ 2023-07-57), and all participants provided written informed consent.

### 2.2. Data collection

Demographic and clinical information, including age, sex, smoking habit, diabetes mellitus (DM), body mass index (BMI), dyslipidemia, hypertension, prior percutaneous coronary intervention (PCI), prior AMI, and medications, were collected. Blood samples were obtained in the morning after overnight fasting (8 h minimum) to determine blood biochemical parameters, including white blood cell (WBC), fasting plasma glucose (FPG), triglyceride (TG), total cholesterol (TC), LDL-C, HDL-C, estimated glomerular filtration rate (eGFR), and UA. The definition of hyperuricemia was serum UA levels >420 µmol/L in men and >360 µmol/L in women (19). The diagnostic criterion for low HDL-C levels was HDL-C< 1.0 mmol/L (20). UHR was defined as the ratio of UA (mg/dl) to HDL-C (mg/dl). The left ventricular ejection fraction (LVEF) was determined during hospitalization using 2D echocardiography. Coronary angiography (CAG) imaging reports were also collected to assess whether vascular occlusion occurred in the left main coronary artery (LM)/multi-vessel and proximal left anterior descending (LAD).

### 2.3. Follow-up

The primary endpoint was major adverse cardiovascular events (MACEs), defined as a combination of death (cardiac and non-cardiac), recurrent MI, revascularization, and readmission (heart failure or angina). The secondary endpoint was all-cause mortality. Follow-up information on hospital discharge was obtained through planned telephone interviews until March 2023. All patients completed follow-ups.

### 2.4. Statistical analysis

Continuous variables were presented as mean ± standard deviation (SD) or median (P25 and P75) and compared using the Mann–Whitney *U* test or Student’s *t*-test as appropriate. The Kolmogorov–Smirnov test was used to check the normality. Categorical variables were expressed as cases (%) and compared with the Chi-square test or Fisher’s exact test accordingly. To assess the biological interaction between HDL-C and UA, we calculated the following indicators after logistic regression analysis: attributable proportion (AP), the relative excess risk to interaction (RERI), and the synergy index (SI) (21). AP and RERI >0 and the confidence interval did not include 0; SI >1 and the confidence interval did not include 1, indicating an interaction and synergistic effect. The RERI and AP were <0 and SI <1, respectively, indicating an interactive and antagonistic effect. The Kaplan–Meier curve was used to draw survival curves, and the log-rank test was performed. Multivariate Cox regression analysis was performed to identify independent predictors of MACE and all-cause mortality. We built three regression models: Model 1 was adjusted for age and sex; Model 2 was the partially adjusted model that was adjusted for variables with *P* < 0.05, including DM, LVEF, TC, FPG, and eGFR, in univariate analysis; and Model 3 was the fully adjusted model that was adjusted for age, sex, BMI, LVEF, current smoking, multi-vessel disease, DM, hypertension, dyslipidemia, prior PCI, prior MI, WBC, FPG, TG, TC, LDL-C, eGFR, STEMI, LM/multi-vessel, proximal LAD, PCI/CABG, antiplatelet drugs, statins, beta-blockers, angiotensin-converting enzyme inhibitors/angiotensin II receptor blockers (ACEI/ARB), UA-lowering drugs, and hypoglycemic drugs. The variance inflation factor (VIF) of the variables included in the models was calculated to avoid deviations caused by multicollinearity. Receiver operating characteristic (ROC) curves were generated, and the area under the curve (AUC) was calculated. All statistical analyses were performed using SPSS 24.0, Excel, and R version 4.1.3. A two-tailed *p*-value of < 0.05 was considered statistically significant.

## 3. Result

### 3.1. Characteristics of the study participants

A total of 480 patients were divided into two groups according to the presence of MACEs. The differences in LVEF, TC, HDL-C, eGFR, and UA levels between the groups were statistically significant. No significant differences were found in other indicators between the two groups (Table 1).

**Table 1:** Baseline characteristics of the study population according to the occurrence of MACE.

Variables	Total (*n* = 480)	Without MACE (*n* = 344)	With MACE (*n* = 136)	*p*-value
Age (years)	64.34 ± 12.57	64.14 ± 12.64	64.86 ± 12.40	0.570
Male, *n* (%)	351 (73.1)	249 (72.4)	102 (75.0)	0.560
BMI (kg/m^2^)	24.03 ± 3.67	23.98 ± 3.78	24.17 ± 3.40	0.611
LVEF (%)	55.53 ± 6.18	56.08 ± 5.70	54.14 ± 7.08	0.005
Risk factors, *n* (%)				
Current smoking	161 (33.5)	114 (33.1)	47 (34.6)	0.767
DM	85 (17.7)	54 (15.7)	31 (22.8)	0.066
Hypertension	257 (53.5)	175 (50.9)	82 (60.3)	0.062
Dyslipidemia	254 (52.9)	187 (54.4)	67 (49.3)	0.314
Prior PCI	29 (6.0)	22 (6.4)	7 (5.1)	0.605
Prior MI	22 (4.6)	16 (4.7)	6 (4.4)	0.910
Laboratory test				
WBC, 109/L	8.40 ± 2.88	8.34 ± 2.73	8.55 ± 3.23	0.491
FPG (mmol/L)	7.17 ± 3.06	7.09 ± 3.08	7.37 ± 3.02	0.371
TG (mmol/L)	1.56 ± 0.80	1.59 ± 0.84	1.50 ± 0.66	0.261
TC (mmol/L)	4.25 ± 1.14	4.33 ± 1.16	4.06 ± 1.06	0.017
LDL-C (mmol/L)	2.66 ± 0.95	2.70 ± 0.98	2.58 ± 0.87	0.216
HDL-C (mmol/L)	1.02 ± 0.27	1.06 ± 0.27	0.93 ± 0.24	<0.001
eGFR (ml/min/1.73m^2^)	95.50 (74.00,108.75)	97.00 (83.00,109.00)	86.00 (59.50,104.75)	<0.001
UA (μmol/L)	368.50 (302.75,446.50)	350.50 (288.00,417.75)	418.00 (351.00,511.00)	<0.001
Initial diagnosis				0.574
STEMI	218 (45.4)	159 (46.2)	59 (43.4)	
NSTEMI	262 (54.6)	185 (53.8)	77 (56.6)	
Angiography findings, *n* (%)				
LM/multi-vessel	333 (69.4)	241 (70.1)	92 (67.6)	0.606
Proximal LAD	180 (37.5)	131 (38.1)	49 (36.0)	0.676
Cardiovascular medications, *n* (%)				
PCI/CABG	365 (76.0)	267 (77.6)	98 (72.1)	0.199
Anti-platelet	443 (92.3)	317 (92.2)	126 (92.6)	0.854
Stains	464 (96.9)	333 (96.8)	131 (97.0)	0.238
Beta-blockers	268 (55.9)	196 (57.0)	72 (53.3)	0.470
ACEI/ARB	241 (50.3)	166 (48.3)	75 (55.6)	0.151
UA-lowering drugs	96 (20.0)	63 (18.3)	33 (24.3)	0.142
Hypoglycemic drugs	85 (17.7)	54 (15.7)	31 (22.8)	0.066

Data were expressed as the mean ± standard deviation or median (interquartile range), *n* (%). *P* values were calculated using the Student’s *t*-test, Mann–Whitney *U* test, Chi-square test, or Fisher’s test accordingly. *P* < 0.05 indicated statistical significance.

BMI, body mass index; LVEF, left ventricle ejection fraction; PCI, percutaneous coronary intervention; MI, myocardial infarction; FH-CAD, family history of coronary artery disease; DM, diabetes mellitus; WBC, white blood cell; FPG, fasting plasma glucose; TC, total cholesterol; TG, triglyceride; LDL-C, low-density lipoprotein-cholesterol; HDL-C, high-density lipoprotein-cholesterol; eGFR, estimated glomerular filtration rate; UA, uric acid; STEMI, ST-segment elevation myocardial infarction; NSTEMI, non-ST-segment elevation myocardial infarction; ACEI, angiotensin-converting enzyme inhibitors; ARB, angiotensin receptor blockers; MACE, major adverse cardiovascular events; Poor CCC, poor coronary collateral circulation.

### 3.2. Interactive effect of UA and HDL-C for MACE and mortality

After logistic regression analysis, according to serum UA and HDL-C levels, the SI was 2.04, indicating that the risk of MACE presenting both risk factors was 1.04 times greater than the sum of the risks exposed to each risk factor alone. The AP showed that a total of 46% of the estimated effect (MACE in patients) was attributable to the interaction between the two biomarkers, implying that when low HDL-C and high UA are present together, the estimated effect is greater than the sum of them alone. However, multiplicative interaction effects were not observed.

Although the contributions of low HDL-C and high UA were negligible, and neither additive nor multiplicative interactions were statistically significant, the risk was significantly correlated when high UA levels were combined with low HDL-C (Table 2).

**Table 2:** Interactive effect of UA and HDL-C for MACE and mortality.

Parameter	MACE HR (95% CI)	*P* value	Death HR (95% CI)	*P* value
Low UA high HDL-C	1 (Reference)		1 (Reference)	
High UA high HDL-C	3.52 (1.78,6.96)	< 0.001	2.20 (0.72,6.77)	0.169
Low UA low HDL-C	3.23 (1.75,5.98)	< 0.001	2.34 (0.87,6.33)	0.094
High UA low HDL-C	10.68 (5.54,20.61)	< 0.001	6.39 (2.43,16.79)	< 0.001
Additive Interaction				
SI	2.04 (1.07,3.88)	< 0.05	2.12 (0.67,6.73)	>0.05
AP	0.46 (0.16,0.76)	< 0.05	0.45 (−0.02,0.91)	> 0.05
REPI	4.93 (−0.37,10.22)	>0.05	2.84 (−1.38,7.07)	>0.05
Multiplicative Interaction				
	0.90 (0.38,2.16)	0.814	1.21 (0.31,4.71)	0.786

Values are presented with Hazard ratios and 95% confidence intervals. *P* < 0.05 indicated statistical significance.

UA, uric acid; HDL-C, high-density lipoprotein cholesterol; MACE, major adverse cardiovascular events; CI, confidence interval; RERI, relative excess risk to interaction; AP, attributable proportion; SI, synergy index.

### 3.3. UHR and MACE

**Figure 1:**
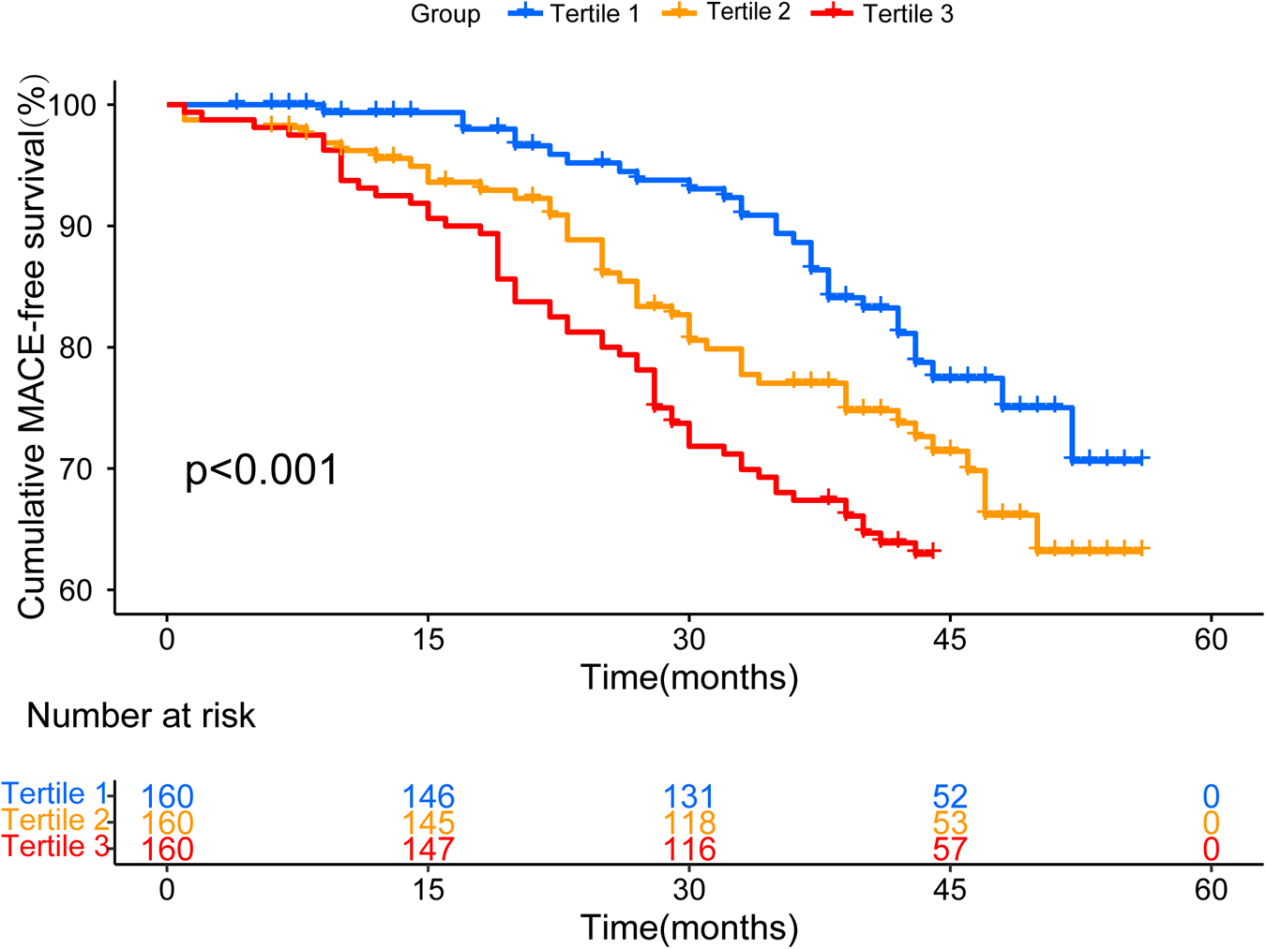
Kaplan–meier survival curve for MACE-free across all UHR tertiles.

The median follow-up time in this study was 41 (30, 46) months. During the follow-up period, 136 MACEs (28.3%) were recorded (Supplementary Table S1). To display the results for patients at different UHR levels, we generated Kaplan-Meier MACE-free plots according to the UHR tertiles (Figure 1). The cumulative incidence of MACE gradually increased with different UHR levels (log-rank test, *P* < 0.001).

**Figure 2:**
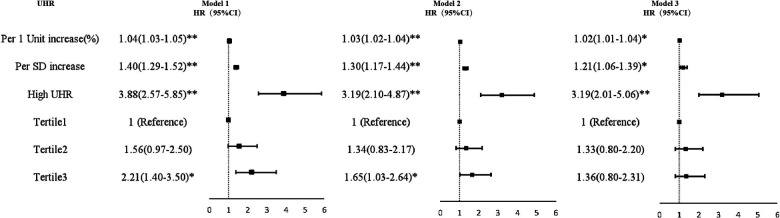
Forest plot of the multivariate Cox regression analyses for the associations between UHR and MACE. Model 1: adjusted for age and sex; Model 2: adjusted for variables with *p*-value < 0.05 in univariate analysis, including DM, LVEF, TC, FPG, and eGFR; Model 3: adjusted for all the variables in Table 1 (except for UA and HDL-C). MACE, major adverse cardiovascular event; HDL-C, high-density lipoprotein-cholesterol; UA, uric acid; UHR, UA to HDL-C ratio; HR, hazard ratio; CI, confidence interval; SD, standard deviation. *p < 0.05. **p < 0.001.

For each SD increase in UHR, the unadjusted HR (95% CI) for MACE was 1.40 (1.29–1.52). Multivariate Cox regression analysis showed that regardless of whether UHR was regarded as a categorical or continuous variable, it was still meaningful after adjusting for confounding factors. In a partially adjusted regression model, for every SD increase in the UHR, the risk of MACE increased by 30% (HR = 1.30; 95% CI 1.17–1.44). UHR was divided into two groups, with an HR of 3.19 (95% CI 2.10–4.87) for MACE in the high UHR group. Compared with patients in the tertile 1, the partially adjusted HR of MACE in the middle and the highest tertile were 1.34 (95% CI 0.83–2.17) and 1.65 (95% CI 1.03–2.64), respectively. The increase in MACE risk from the first to the third level was statistically significant (*P* for trend = 0.038). Similar patterns were observed in the fully adjusted model (Figure 2).

**Figure 3:**
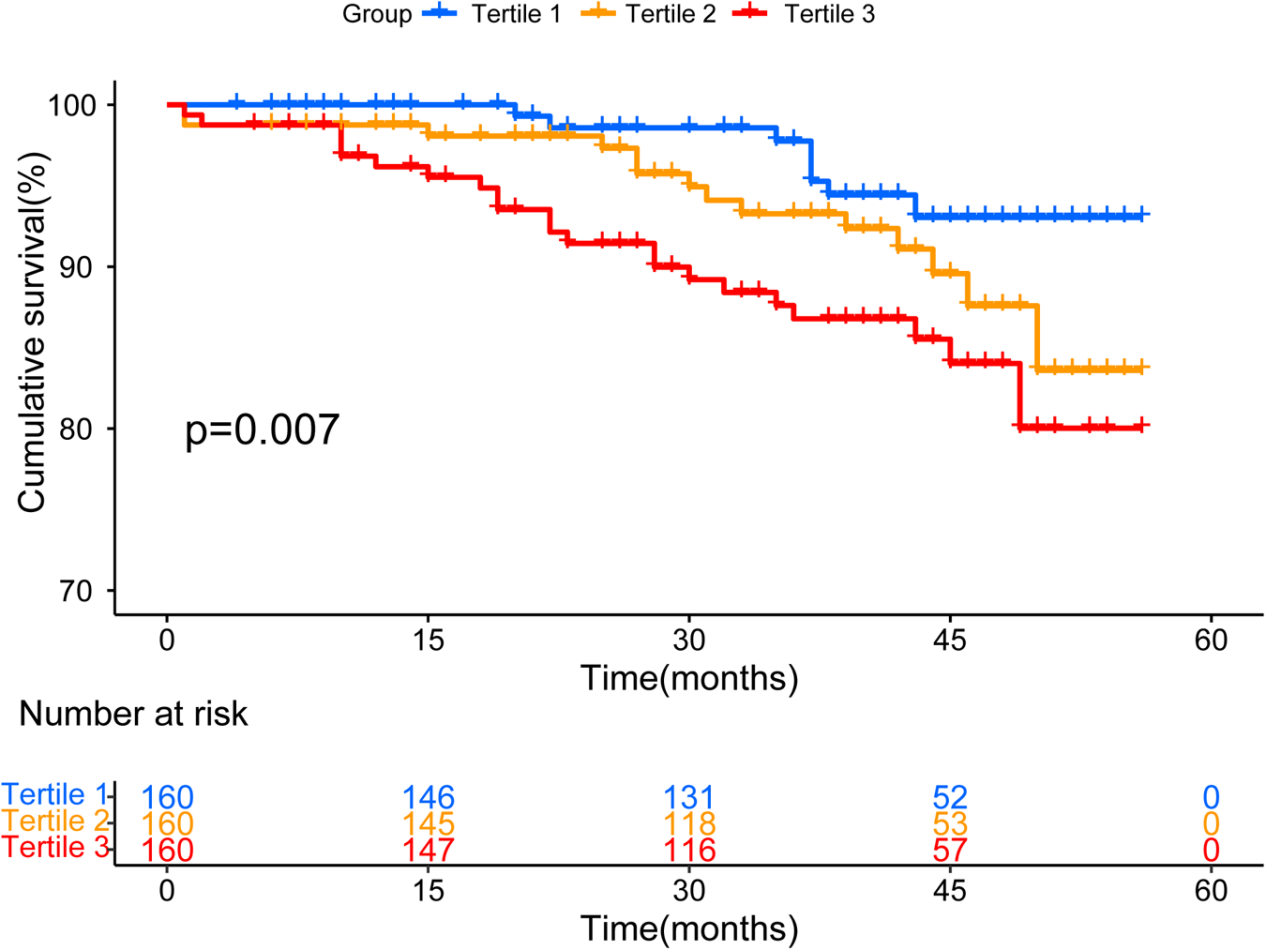
Kaplan–meier survival curve for survival across all UHR tertiles.

We further investigated the association between UHR status and all-cause mortality. The cumulative incidence of survival gradually increased with different UHR levels (log-rank test, *P* = 0.007) (Figure 3). UHR was also an independent risk factor of all-cause mortality, especially as a continuous variable (Supplementary Table S2).

### 3.4. ROC analysis

**Figure 4:**
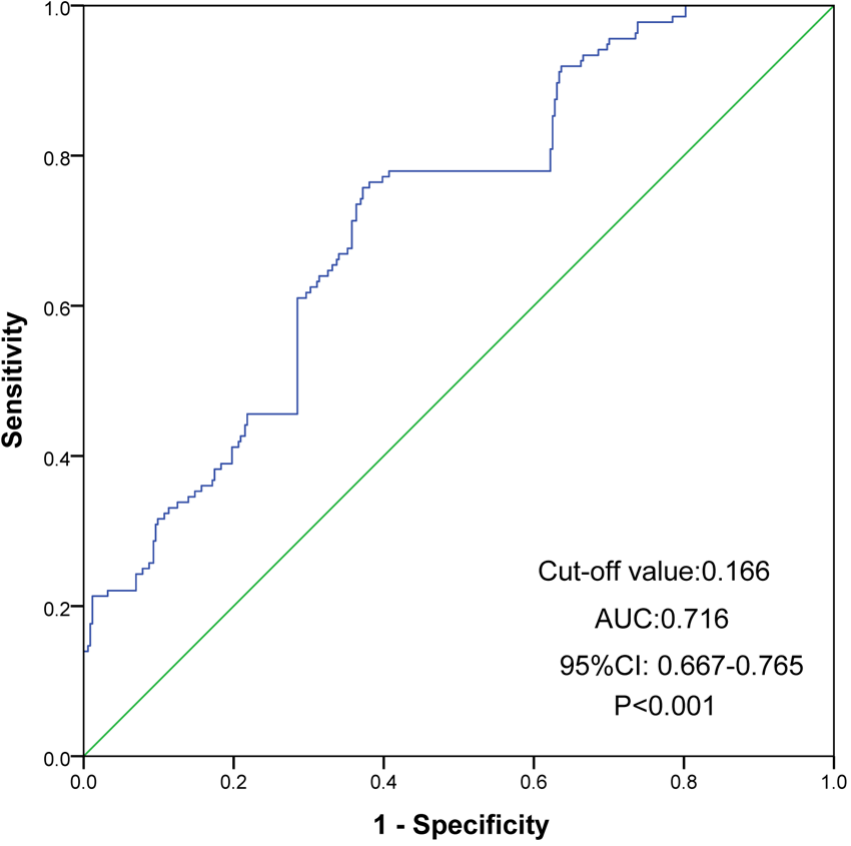
ROC curves of UHR for the prediction of MACE. The cutoff value of UHR was 0.166 and the AUC was 0.716 (95% CI 0.667 to 0.765, *P* < 0.001). ROC, receiver operating characteristic; MACE, major adverse cardiovascular event; UHR, uric acid to high-density lipoprotein cholesterol ratio; CI, confidence interval; AUC, area under ROC curve.

**Figure 5:**
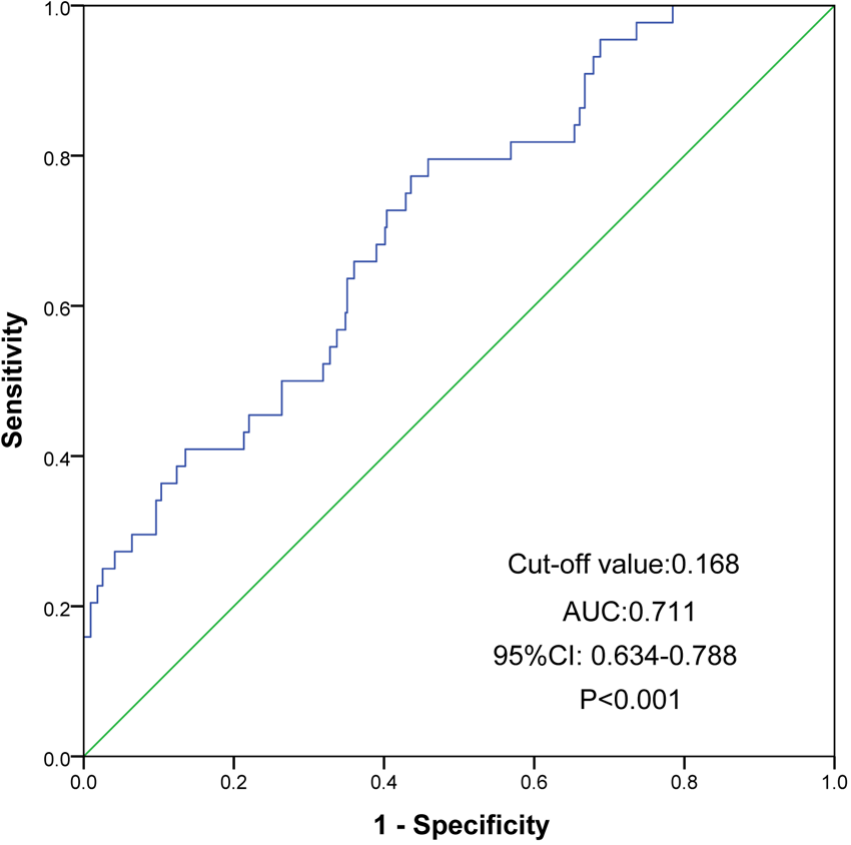
ROC curves of UHR for the prediction of mortality. The cutoff value of UHR was 0.168 and the AUC reached 0.711 (95% CI 0.634 to 0.788, *P* < 0.001). ROC, receiver operating characteristic; UHR, uric acid to high-density lipoprotein cholesterol ratio; CI, confidence interval; AUC, area under ROC curve.

ROC analysis was performed to further assess the prognostic value and predictive performance of UHR. For MACE, the area under the curve (AUC) was 0.716 (95% CI 0.667–0.765, *P* < 0.001) (Figure 4). For all-cause mortality events, the AUC was 0.711 (95% CI 0.634–0.788, *P* < 0.001) (Figure 5).

## 4. Discussions

To the best of our knowledge, our study is the first to investigate the prognostic impact of the interaction between UA and HDL cholesterol levels in AMI. We investigated the predictive value of UHR for AMI prognosis. Our study found that the interaction between UA and HDL enhances prognosis prediction and that UHR is a simple and easily available prognostic indicator independent of traditional risk factors.

UA and HDL-C, which are biomarkers of oxidative stress, inflammation, and metabolism, showed a positive interaction that increased the risk of mortality and poor cardiovascular prognosis in patients with AMI, an effect that was greater than their sum. UA, an end product of purine metabolism, is a potent antioxidant that may protect against oxidative stress (3). However, studies have shown that UA shifts from antioxidant effects to pro-oxidant effects depending on its level (4). High UA levels activate the renin-angiotensin system (RAS) and reduce insulin-induced nitric oxide (NO) synthesis in endothelial cells (22, 23). In addition, high UA levels promote insulin resistance in adipocytes (24). Controversies and pressing questions remain regarding the risks and prognoses of UA and CVD. The relationship between UA level and CVD prognosis has been reported to be linear and non-linear (25). In addition, studies with subgroup analyses have found that UA has different effects on cardiovascular prognosis in different populations, such as those with different ages and risk factors (7). Several studies have shown that elevated UA levels independently predict adverse clinical outcomes after AMI, including increased mortality, morbidity, and MACE (26, 27). In addition, a recent study reported that high UA levels, even those within the normal range, may be a risk factor for mortality in patients with AMI treated with primary PCI (28). However, UA may also have a protective effect owing to its antioxidant properties, which may explain some of the inconsistent findings in AMI.

HDL-C is an important component of lipid metabolism. Previous studies have reported its protective effect against CVD (8). Lower HDL-C levels are associated with a higher risk of CVEs and more severe atherosclerosis, even in patients with low LDL-C levels (29). In addition, several studies have shown that low HDL-C levels in patients with AMI can predict MACEs (8, 30). The protective effect of HDL-C is thought to be related to reverse cholesterol transport by macrophages (31). Furthermore, HDL-C was recently found to promote endothelial homeostasis by increasing NO production and inhibiting key pathways involved in endothelial cell apoptosis and vascular inflammation (32). As studies have progressed, it has been observed that higher HDL-C levels are not necessarily protective against CVD and that very high levels may even be detrimental (10). Emerging studies suggest that raising HDL-C levels may not reduce future CVEs and atherosclerotic burden (33). Additionally, extreme elevations in HDL-C levels may indicate alterations in HDL-C particles in certain individuals, which may accelerate CVD progression (34). For HDL-C, it has been suggested that the impact of its subclasses and function on CVD should be explored in depth. The modification or enhancement of HDL-C levels by other risk factors should not be ignored. The metabolism and regulation of HDL-C are far more complex than previously thought and are subject to changes in specific situations, such as disease progression (35). There is still controversy regarding HDL-C and CVD risk and prognosis, which requires further mechanistic and clinical studies.

A previous study has reported a significant correlation between high UA and low HDL-C levels. A mechanistic study suggested that low serum HDL-C and hyperuricemia may have synergistic adverse effects on the cardiovascular system through oxidative damage to endothelial cells and insulin resistance (11, 12). In resource-limited settings, interaction analysis can identify patients who would benefit the most from a given intervention. However, to date, no study has explored the additive interactions between UA and HDL-C in AMI. The use of SI >1, RERI >0, and AP >0 indicated the presence of biological interactions (36). AP confirmed that 46% of MACEs could be attributed to the interaction between low HDL-C and high UA, which revealed that we could significantly improve the poor prognosis of dual-exposure patients by changing one of the risk factors: SI >1 and the confidence interval did not include 1, indicating an interaction and a synergistic effect. Our study found that patients with high and low HDL-C levels had a worse prognostic risk. This is important because the additive interaction may help to determine which patients will benefit the most from the intervention, which will help in the precise management of residual risk to improve the prognosis of AMI.

UHR is a new inflammatory and metabolic index of combined UA and HDL cholesterol that correlates with the level of glycemic control and complications, metabolic syndrome, NAFLD, ischemic cardiomyopathy, and the degree of hypertension control (15, 37–39). Recently, UHR was found to predict CVD mortality in patients undergoing peritoneal dialysis (40). Li et al. found that UHR predicts functional stenosis in patients with CAD (41). However, no previous study has explored the relationship between UHR status and MACE in the real world. In this study, we report, for the first time, that high UHR is associated with increased adverse clinical events in patients with AMI. The prognostic value may be due to an increase in UA and a decrease in HDL-C levels. Moreover, the additive interaction could partly explain the value of our observed UHR for the prognosis of AMI. In our study, the mean UHR level was significantly higher in AMI patients (17.70 ± 8.75%) than in the general population (10.08 ± 4.22%) (17). This is not unexpected, as patients with AMI as advanced CAD usually have lower HDL-C and higher UA levels. UHR is a simple and efficient prognostic biomarker for AMI, and its mechanism and application require further investigation.

This study had a few limitations. First, as a single-center retrospective cohort study, it may be biased, and the sample size is relatively small; therefore, we used potential confounding variables as covariates in the regression model to control for referral bias and possible confounding factors. Second, we only analyzed baseline clinical data and could not measure the effect of UA and HDL-C variations over time on prognosis. Third, we did not record dietary habits or other behaviors that might affect the UA and HDL-C levels. Finally, because the participants in our study were Chinese only, the present results may not apply to other ethnic groups. Future large-sample, multicenter, and well-designed prospective studies are needed to strengthen our conclusions.

## 5. Conclusion

In conclusion, the interaction between UA and HDL has a synergistic effect and provides further information for risk stratification of patients with AMI. UHR is a simple and easily available prognostic indicator independent of traditional risk factors.

## Data Availability

The raw data supporting the conclusions of this article will be made available by the authors, without undue reservation.
