# Supplementary material for: The interaction between uric acid and high-density lipoprotein cholesterol on the prognosis of patients with acute myocardial infarction
Source: Front Cardiovasc Med. 2023 Jul 10;10:1226108. doi: 10.3389/fcvm.2023.1226108 (PMC10363914; doi:10.3389/fcvm.2023.1226108)
Supplement: Supplementary file 1 [file Table1.doc]

**Supplement Table 1** Follow up result according to the tertiles of the UHR

| Variables | Total(n=480) | T1(n=160) | T2(n=160) | T3(n=160) | p-value |
| --- | --- | --- | --- | --- | --- |
| MACE | 136(28.3) | 30(18.8) | 44(27.5) | 62(38.8) | <0.001 |
| All-cause death | 44(9.2) | 8(5.0) | 14(8.8) | 22(13.8) | 0.025 |
| Cardiovascular death | 38(7.9) | 8(5.0) | 10(6.3) | 20(12.5) | 0.029 |
| recurrent MI | 28(5.8) | 3(1.9) | 10(6.3) | 15(9.4) | 0.016 |
| revascularization | 47(9.8) | 12(7.5) | 12(7.5) | 23(14.4) | 0.058 |
| Re-hospital | 79(16.5) | 19(11.9) | 27(16.9) | 33(20.6) | 0.106 |

MACE, major adverse cardiovascular events;MI,myocardial infarction.
