# Supplementary material for: The interaction between uric acid and high-density lipoprotein cholesterol on the prognosis of patients with acute myocardial infarction
Source: Front Cardiovasc Med. 2023 Jul 10;10:1226108. doi: 10.3389/fcvm.2023.1226108 (PMC10363914; doi:10.3389/fcvm.2023.1226108)
Supplement: Supplementary file 2 [file Table2.doc]

Supplement Table2 Multi-variable Cox regression analyses for the associations between UHR and Mortality

| UHR | HR(95% CI) | | |
| --- | --- | --- | --- |
|  | Model 1 | Model 2 | Model 3 |
| Per 1 Unit increase(%) | 1.05(1.04-1.07)** | 1.04(1.02-1.05)** | 1.04(1.01-1.07)* |
| Per SD increase | 1.54(1.36-1.74)** | 1.35(1.16-1.57)** | 1.39(1.08-1.80)* |
| High UHR | 4.32(2.03-9.22)** | 2.82(1.32-6.02)* | 2.17(0.94-5.02) |
| Tertile1 | 1 (Reference) | 1 (Reference) | 1 (Reference) |
| Tertile2 | 1.78(0.74-4.29) | 1.03(0.41-2.59) | 0.82(0.31-2.19) |
| Tertile3 | 2.72(1.17-6.33)* | 1.39(0.58-3.33)* | 0.84(0.30-2.32) |
| P for trend | 0.018 | 0.373 | 0.792 |

Model 1: adjusted for age and gender

Model 2: adjusted for variables with p-value < 0.05 in univariate analysis, including Age,DM, LVEF,eGFR, and Beta-blockers.

Model 3: adjusted for all the variables in table 1(except for UA and HDL-C).

Abbreviations: HR, hazard ratio; CI, confidence interval; SD, standard deviation

* p < 0.05

** p < 0.001
